# Impact of Peri‐Implant Phenotype on Implant Therapy Outcomes: A 5‐Year Cohort Analysis on Soft Tissue—Level Implants

**DOI:** 10.1111/jcpe.70062

**Published:** 2025-12-12

**Authors:** Hamoun Sabri, Parham Hazrati, Lorenzo Tavelli, Carlos Garaicoa‐Pazmino, Javier Calatrava, Hom‐Lay Wang, Shayan Barootchi

**Affiliations:** ^1^ Department of Periodontics and Oral Medicine University of Michigan School of Dentistry Ann Arbor Michigan USA; ^2^ Center for Clinical Research and Evidence Synthesis in Oral Tissue Regeneration (CRITERION) Ann Arbor Michigan USA; ^3^ Division of Periodontology, Department of Oral Medicine, Infection, and Immunity Harvard School of Dental Medicine Boston Massachusetts USA; ^4^ Department of Periodontics University of Iowa College of Dentistry and Dental Clinics Iowa City Iowa USA; ^5^ School of Dentistry, Universidad de Especialidades Espiritu Santo Samborondon Ecuador; ^6^ Section of Graduate Periodontology, Faculty of Odontology University Complutense Madrid Spain

**Keywords:** bone‐level implants, marginal bone loss, one‐piece implant, peri‐implant health, peri‐implant mucositis, peri‐implant soft‐tissue deficiency, peri‐implantitis, tissue‐level implants, transmucosal implant

## Abstract

**Objective:**

To investigate the associations between peri‐implant phenotype characteristics and long‐term outcomes of soft tissue–level implants.

**Methods:**

Twenty‐five tissue‐level implants from a previous controlled clinical trial were evaluated at 1‐ and 5‐year follow‐ups after crown delivery. Data included ultrasonographic scans (mucosal thickness and supracrestal tissue height), standardised 2D radiographs, cone beam computed tomography, clinical outcomes (mucosal recession, probing pocket depth, bleeding on probing), patient‐reported outcomes and peri‐implant health status. Standard logistic and linear regression models were used to analyse associations of implant‐ and patient‐related factors with outcomes, including peri‐implant disease status, mucosal recession and marginal bone level (MBL) changes.

**Results:**

Five‐year implant survival was 100%, with peri‐implant mucositis diagnosed in 36% of the implants. Mean MBL changes from the first to the fifth year was 0.29 ± 0.29 mm. Baseline (pre‐surgical) buccal soft‐tissue thickness < 1.5 mm (OR: 5.20, *p* = 0.007) and increased plaque scores (OR: 30.11, *p* < 0.001) were associated with peri‐implant mucositis, whereas buccal bone thickness ≥ 1.5 mm (OR: 0.48, *p* = 0.039), mucosal thickness around implant (OR: 0.20, *p* = 0.001) and supracrestal tissue height (OR: 0.50, *p* = 0.002) emerged as protective factors. Mucosal recession was significantly associated with baseline buccal soft‐tissue thickness < 1.5 mm (estimate = 0.27, *p* = 0.03) and keratinised mucosa width of < 2 mm (estimate = 0.39, *p* = 0.021). Clinical thresholds associated with long‐term peri‐implant health were defined as follows: supracrestal tissue height ≥ 2.8 mm, restorative emergence angle ≤ 35.5°, mucosal thickness 1.8 mm and buccal bone thickness 1.5 mm. Patients reported a high overall satisfaction (visual analogue scale: 88.2%). Colour Doppler ultrasonography showed a strong correlation between tissue perfusion and peri‐implant disease (*r* = 0.93, *p* < 0.001).

**Conclusions:**

Within the limitations of this study, tissue‐level implants showed excellent survival rates and patient satisfaction at 5 years. Several peri‐implant parameters—such as supracrestal tissue height ≥ 2.8 mm, restorative emergence angle ≤ 35.5° and buccal bone thickness ≥ 1.5 mm—were associated with favourable outcomes. These exploratory thresholds may be considered hypothesis‐generating and could help guide future research and clinical monitoring, although validation in larger cohorts is essential.

**Trial Registration:** www.clinicaltrials.gov/study/NCT02925078

## 1 Introduction

As dental implants have become the gold standard for rehabilitating edentulous patients, long‐term success of implants remains a critical focus. Despite advancements, achieving and maintaining stability continues to pose challenges (Berglundh et al. 2018; French et al. 2021; Monje et al. 2023). Recent research underscores the pivotal role of the peri‐implant phenotype—comprising soft‐ and hard‐tissue characteristics—in implant health and success (Monje et al. 2023; Avila‐Ortiz et al. 2020; Önder and Alpaslan 2024).

The soft‐tissue height/supracrestal tissue height (STH), supracrestal tissue adhesion, mucosal thickness (MT), keratinised mucosa width (KMW) and peri‐implant bone thickness are critical components of the peri‐implant phenotype that influence the long‐term outcomes of dental implants (Monje et al. 2023; Avila‐Ortiz et al. 2020; Galarraga‐Vinueza and Tavelli 2023). STH, the vertical soft‐tissue height of the peri‐implant mucosa, is established as a physiological adaptation to the transmucosal component and plays a significant role in maintaining marginal bone level (MBL) stability (Tavelli et al. 2021). Inadequate STH (< 3 mm) often results in early bone remodelling, especially with juxtacrestal bone level implants, while excessive STH (> 3 mm) has been linked to delayed mucositis resolution and higher peri‐implant disease risk (Berglundh and Lindhe 1996; Chan et al. 2019). On the other hand, MT has been shown to correlate positively with MBL stability and peri‐implant health. Evidence suggests that a minimum MT of 2 mm, particularly in the cervical region, is essential for predictable aesthetic and functional outcomes (Suárez‐López Del Amo et al. 2016; Thoma et al. 2018; Barootchi, Rodriguez, et al. 2025). Ultimately, a KMW of 2 mm is pivotal for maintaining peri‐implant health over time. While patients with optimal oral hygiene may not require a wide band of keratinised mucosa, a recent meta‐analysis on nearly 2000 dental implants indicated an odds ratio (OR) of 1.8 and 4.05 for radiographic bone loss and mucosal recession (MRec), respectively, around implants with < 2 mm of KMW (Sabri et al. 2025). Furthermore, a recent long‐term (10‐year) study indicated that a lack of KMW (0 mm) is associated with a higher prevalence rate of peri‐implant diseases compared to sites with > 0 mm of KMW (Mancini et al. 2024). Taken together, these findings suggest that while the exact threshold remains debatable, KMW is generally recognised as a crucial clinical parameter.

Peri‐implant bone thickness further affects stability. Bone adapts to establish the STH required for implant support, often leading to early remodelling in sites lacking adequate hard or soft tissue. Thus, balancing tissue dimensions is vital. Implant placement should be prosthetically guided to achieve optimal STH, MT and KMW while limiting remodelling and inflammation (Spinato et al. 2022).

Modern implants are categorised as bone level or soft‐tissue level (STL) implants. STL implants, which heal transgingivally, reduce surgical steps and preserve MBL by minimising microgaps and micromovements (Buser et al. 1999; Sasada and Cochran 2017; Chacun et al. 2024). Their design supports a stable soft‐tissue seal that is crucial for protecting the underlying bone (Salvi et al. 2015). Pre‐clinical and clinical studies show that the STL surface characteristics promote integration and long‐term stability (Schwarz et al. 2007; Tomasi et al. 2014; Barootchi, Arefi, et al. 2025). However, longitudinal evidence specifically linking STL outcomes with patient factors, tissue dynamics and satisfaction remains scarce.

Thus, the present 5‐year study explored the clinical, radiographic, ultrasonographic and patient‐reported outcomes of STL implants and their association with peri‐implant tissue characteristics.

## 2 Materials and Methods

### 2.1 Study Design

The present study was conceptualised as a 5‐year longitudinal cohort analysis evaluating the impact of peri‐implant phenotype characteristics on implant therapy outcomes. The analysis was based on follow‐up investigation from a previous prospective controlled clinical trial conducted between November 2016 and December 2019 on the impact of mucosal phenotype on MBL around STL implants (HUM00095933 and NCT02925078) (Garaicoa‐Pazmino et al. 2021). For the present secondary analysis, we specifically investigated how peri‐implant phenotype influenced long‐term clinical and radiographic outcomes. The protocol of the current follow‐up analysis was also approved by the Institutional Review Board of the University of Michigan Medical School (HUM00194618) and is in accordance with the Declaration of Helsinki of 1975, revisited in Fortaleza in 2013 (World Medical Association 2013). The STROBE (STrengthening the Reporting of OBservational studies in Epidemiology) statement was followed for preparing the present manuscript (see Data S1). (Vandenbroucke et al. 2014).

### 2.2 Participants

In the previous prospective controlled trial, participants were enrolled based on pre‐surgical (baseline) vertical soft‐tissue thickness (STT) and classified into thin (≤ 2 mm) and thick (> 2 mm) phenotype groups measured via trans‐gingival probing at the centre of the edentulous ridge (Garaicoa‐Pazmino et al. 2021). In the previous trial, 26 patients (13 in each group) completed the 1‐year examination following crown delivery (CD). For the present long‐term secondary assessment, all patients were pooled into a single cohort to explore the impact of peri‐implant phenotype parameters on outcomes over the extended follow‐up period. A complete description of eligibility criteria is available in the original publication (Garaicoa‐Pazmino et al. 2021) as well as in Data S1.

All participants who completed the original 1‐year study were invited to undergo a 5‐year follow‐up assessment. Figure S1 depicts the study flow‐chart.

### 2.3 Interventions

The complete interventional/surgical protocol has been previously reported in detail (Garaicoa‐Pazmino et al. 2021) (see Data S1 for details).

#### 2.3.1 Data Collection and Clinical Parameters

At the 5‐year visit, the following clinical parameters were collected by an examiner who was blinded to the initial group of the subjects:

- Probing pocket depths (PPDs): This was measured using a UNC‐15 periodontal probe on six sites (mesio‐buccal, mid‐buccal, disto‐buccal, mesio‐lingual, mid‐lingual and disto‐lingual). The mean value of these six sites was calculated.
- Mucosal recession (MRec): MRec was defined as the amount of mid‐buccal apical shift of the peri‐implant mucosal margin (in millimetres) at the 5‐year appointment compared to its position at 1‐year post loading. (MRec at 5 years = Mucosal level at the fifth year – mucosal level at the first year post loading).
- KMW, Modified Plaque Index (mPI), Bleeding on Probing (BoP) and Suppuration (SUP): See Data S1 for a detailed description.

The following clinical and radiographic data were also used from the initial study data:

- Pre‐surgical (baseline) buccal and vertical soft‐tissue thickness (STT): measured using a calliper intra‐surgically after flap elevation.

#### 2.3.2 Radiographic Assessment

**FIGURE 1:**
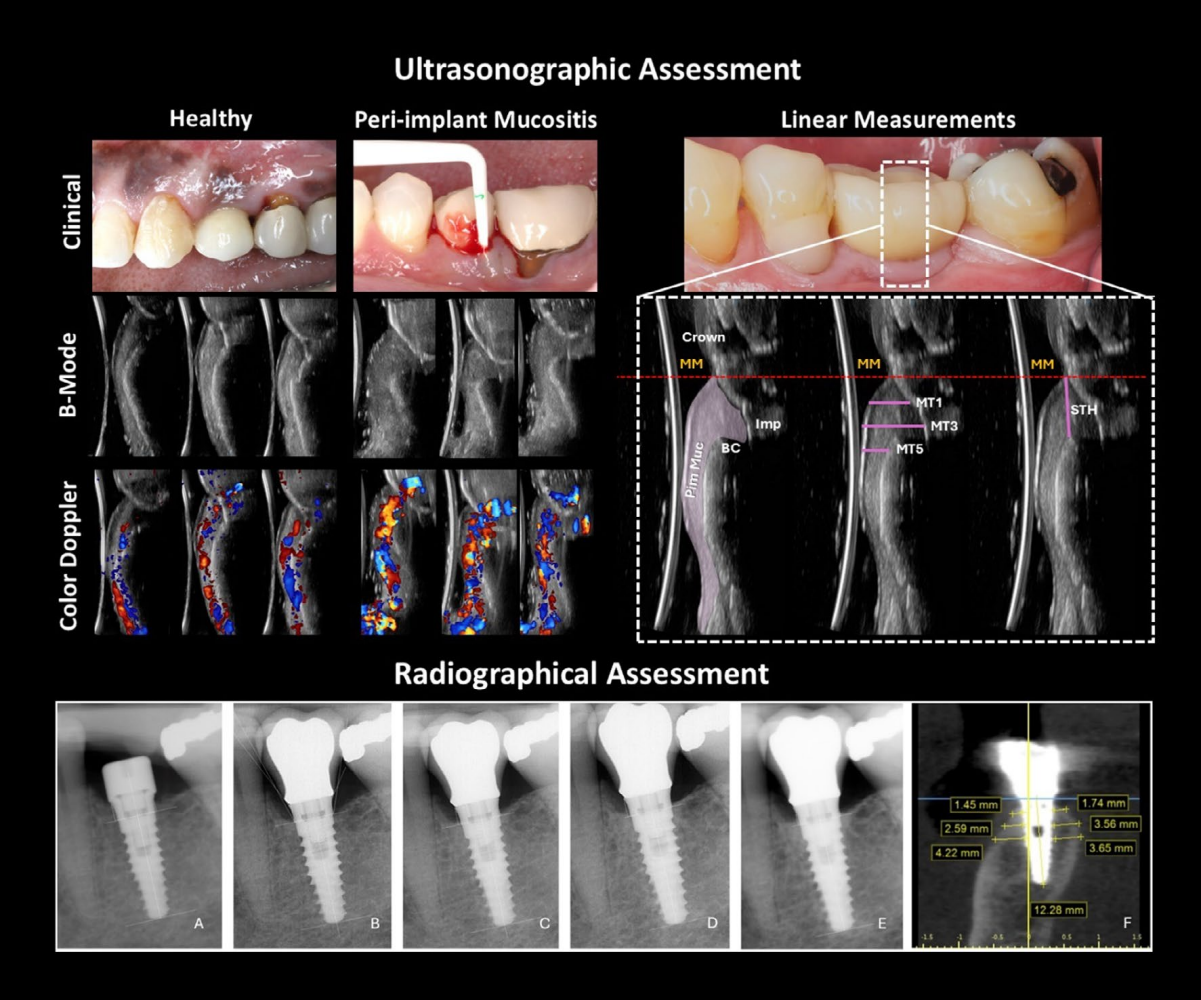
(top left): Clinical, B‐mode and C‐mode ultrasonographic images of a healthy and diseased implant. (top right): Ultrasonographic measurements. The red‐dashed line demarcates the mucosal margin (MM) around the implant. Peri‐implant mucosa (Pim Muc) is outlined as the pink area. Mucosal thickness (MT) was measured at 1 (MT1), 3 (MT3) and 5 (MT5) mm from the MM. Supracrestal tissue height (STH) was defined as the distance between MM and peri‐implant bone crest (BC). (Bottom): Standardised radiographic measurements. Peri‐apical x‐rays from baseline (A), crown delivery (B), 1 year (C), 3 years (D) and 5 years (E). The yellow lines show marginal bone level as well as emergence angle measurements. Buccal and labial bone thickness assessment from CBCT images (F). Radiographic assessments included standardised periapical radiographs with stent guidance and CBCT scans. Marginal bone‐level (MBL) changes were calculated from mesial and distal measurements (ImageJ), with high intra‐examiner reliability (ICC = 0.92). Three‐dimensional CBCT analysis quantified buccal and palatal bone thickness at 2, 4 and 6 mm below the implant platform.

Figure 1 (bottom) depicts the radiographic assessment performed (see Data S1 for a detailed description). Radiographic examinations were performed by one calibrated examiner on standardised intraoral periapical images. Measurement error was assessed as the absolute difference (Alibegovic et al. 2025) between repeated assessments on 10 non‐study implant radiographs, resulting in a mean error of 0.19 ± 0.21 mm and intra‐examiner ICC of 0.92 (95% CI: 0.88–0.96). Moreover, buccal and palatal bone thickness (BBT, PBT) were measured from CBCT scans, and the restorative emergence angle (mesial and distal) was calculated from standardised intra‐oral x‐rays.

#### 2.3.3 Ultrasonographic Examination

See Data S1 for complete description. A pre‐calibrated, blinded examiner (S.B.) performed ultrasonographic image acquisition (the calibration phase protocol is provided in Data S1). The complete ultrasonographic assessment protocol has been described in previous publications (Barootchi et al. 2022; Galarraga‐Vinueza et al. 2024). On the mid‐facial ultrasound scans, the following variables were measured (Figure 1; top right):

- MT: measured at 1, 3 and 5 mm below the mucosal margin. A mean value was considered for each implant for the analysis; this corresponds to the thickness of soft tissue after implant restoration.
- STH: measured as the linear distance from mucosal margin to the bone crest (Avila‐Ortiz et al. 2020), which corresponds to the vertical supracrestal component of the soft tissue after implant restoration.

Additionally, the colour‐Doppler mode was used to quantify the tissue perfusion (in colour velocity [CV]) (Figure 1; top left).

#### 2.3.4 Patient‐Reported Outcome Measures (PROMs)

At the end of the 5‐year follow‐up visits, PROMs were assessed using a standardised questionnaire filled out by patients. The PROMs assessed consisted of overall satisfaction via a visual analogue scale (VAS), aesthetic and functional perception and OHIP‐14 questionnaire. For a detailed description of PROMs assessment, see Data S1.

### 2.4 Study Outcomes

At the 5‐year recall, peri‐implant disease status and implant survival were set as the primary outcomes of the study. Peri‐implant health was defined according to 2017 AAP/EFP World Workshop criteria (Berglundh et al. 2018) as health, peri‐implant mucositis and peri‐implantitis using clinical and radiographic parameters, with peri‐implantitis diagnosis requiring ≥ 2 mm bone loss to account for remodelling and measurement error. Moreover, survival in this study was defined as a functionally integrated implant at the time of assessment.

Secondary outcomes of the study included the following:

- MBL changes.
- Peri‐implant MRec.
- PROMs, including patient satisfaction, aesthetic scores and OHIP‐14.
- Ultrasonographic tissue perfusion (CV).

### 2.5 Statistical Analysis

All statistical analyses were performed by one author with experience in biostatistics (H.S). A full description of the tests performed is provided in Data S1.

## 3 Results

### 3.1 Descriptive Statistics and Study Population

**FIGURE 2:**
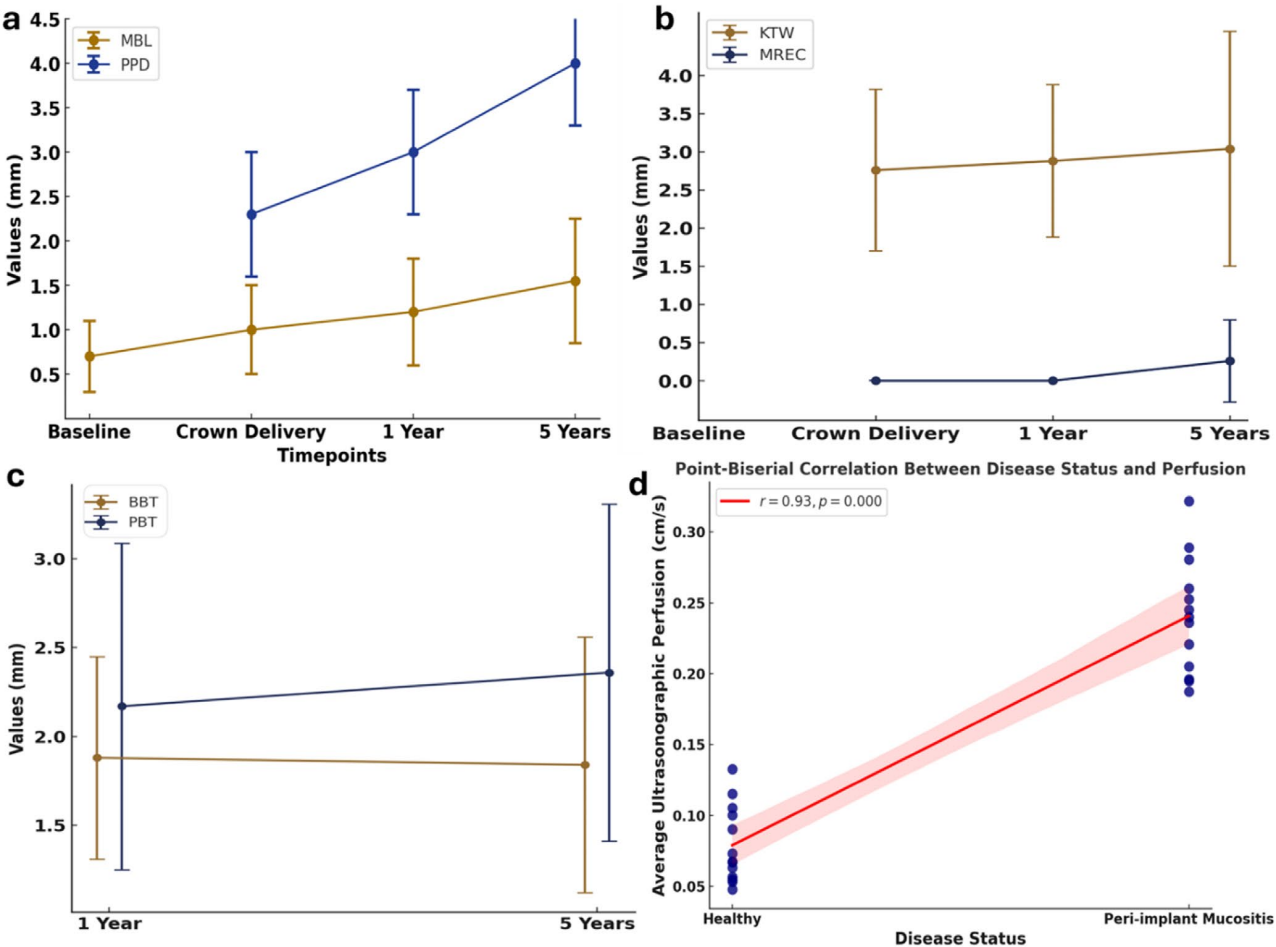
(a–c) Changes of peri‐implant phenotype and health‐related variables over time. (d) Graph depicting the results of point biserial correlation analysis of tissue perfusion and peri‐implant health status.

At the 5‐year follow‐up, 25 patients (25 implants) completed the study, with one dropout compared to the 1‐year phase. The 5‐year survival rate of the implants was 100%. Nine implants were diagnosed with peri‐implant mucositis (36%) and 16 (64%) with peri‐implant health. Given the measurement error established in our study (0.19 ± 0.21 mm) and the observed MBL changes (mean 0.56 ± 0.39, maximum: 1.33 mm), this confirms that all implants would consistently remain within the peri‐implant health or mucositis categories, with none shifting into peri‐implantitis. The complete results of descriptive analysis and changes in the variables over time are provided in the Appendix and summarizsd in Table 1 and Figure 2a–c.

**TABLE 1:** (Top): Study subject characteristics who completed 5‐year follow‐up assessments. (Middle): Changes in clinical variables over study timeline. (Bottom): Ultrasonographic color doppler and tissue perfusion in different sites aroudn implants.

Variable	Value
Number of subjects/implants	25/25
Age (mean ± SD)	54.20 ± 12.01
Gender (M/F) (%)	
Male	15 (60%)
Female	10 (40%)
Implant location	
Maxilla (*n*) (%)	9 (36%)
Mandible (*n*) (%)	16 (64%)
Implant site	
Premolar (*n*) (%)	11 (44%)
Molar (*n*) (%)	14 (56%)
Implant length	
9 (*n*) (%)	3 (12%)
10.5 (*n*) (%)	20 (80%)
12 (*n*) (%)	2 (8%)
Implant diameter	
3.8 (*n*) (%)	17 (68%)
4.6 (*n*) (%)	8 (32%)
Survival rate (%)	100%
Peri‐implant health status	
Healthy (*n*) (%)	16 (64%)
Peri‐implant mucositis (*n*) (%)	9 (36%)
Restorative emergence angle	
Mesial (degrees)	36.88 ± 7.21
Distal (degrees)	35.19 ± 10.98
Average	36.04 ± 8.41
BoP	
No (*n*) (%)	6 (24%)
Spot (*n*) (%)	10 (40%)
Profuse (*n*) (%)	9 (36%)
mPI	
0	10
1	7
2	8
Compliance with supportive therapy	
Non‐compliance (no supportive therapy)	0
Erratic compliance (< 2 supportive therapy/year)	14
Regular compliance (≥ 2 supportive therapy/year)	11
Mucosal thickness after crown delivery (mm)	1.87 ± 0.40
Supracrestal tissue height (mm)	3.26 ± 0.70
Vertical soft‐tissue thickness (baseline)	2.16 ± 0.53
Buccal soft‐tissue thickness (baseline)	0.84 ± 0.39

	Timepoints				Changes between time points			
Variable	BL^a^	CD	1Y	5Y	BL‐CD	CD‐1Y	1Y‐5Y	CD‐5Y
MBL	0.78 ± 0.38	1.01 ± 0.47	1.28 ± 0.42	1.57 ± 0.42	0.23 ± 0.22 [0–0.9]	0.27 ± 0.25 [−0.09–1.09]	0.29 ± 0.29 [−0.01–1.15]	0.56 ± 0.39 [−0.06–1.33]
PPD	NR	2.31 ± 0.41	3.26 ± 0.49	3.96 ± 0.51	NR	0.95 ± 0.65 [−0.33–2.17]^b^	0.70 ± 0.75 [−0.7‐2.53]	1.65 ± 0.63 [0.63–3.03]^b^
BBT	NR	NR	1.88 ± 0.57	1.84 ± 0.72	NR	NR	−0.03 ± 0.81	NR
PBT	NR	NR	2.17 ± 0.92	2.36 ± 0.95	NR	NR	0.19 ± 0.07	NR
KMW	NR	2.76 ± 1.06	2.88 ± 1.00	3.04 ± 1.54	NR	0.12 ± 0.46	0.15 ± 1.36	0.27 ± 1.34
MRec	NR	0.00 ± 0.00	0.00 ± 0.00	0.26 ± 0.54	NR	0 ± 0	0.26 ± 0.54 [−1.33–1.33]	0.26 ± 0.54 [−1.33–1.33]

	Ultrasonographic tissue perfusion					
	Healthy			Peri‐implant mucositis		
	Mesial	Mid‐buccal	Distal	Mesial	Mid‐buccal	Distal
Colour Doppler (cm/s)	0.071 ± 0.042	0.079 ± 0.041	0.080 ± 0.032	0.263 ± 0.092	0.224 ± 0.082	0.207 ± 0.089
*p‐*value*				< 0.01	< 0.01	0.02

Abbreviations: 1Y, 1 year follow‐up; 5Y, 5‐year follow‐up; BBT, buccal bone thickness; BL, baseline (implant surgery); BoP, bleeding on probing; CD, crown delivery; F, female; KMW, keratinised mucosa width; M, male; MBL, marginal bone level; MRec, mucosal recession; NR, not recorded; PBT, palatal bone thickness; PPD, probing pocket depth; SD, standard deviation.

^a^ Baseline: implant placement surgery.

^b^ Significant with either paired *t*‐test or Wilcoxon signed‐rank test based on the normality of data.

* *p*‐value of comparison of each site with its other counterpart (healthy vs. diseased).

### 3.2 Peri‐Implant Disease Status and Associated Factors

Table 2 presents the results of the logistic regression analysis on peri‐implant disease status. In the univariable regression analysis, several factors were significantly associated with disease (gender, pre‐surgical buccal STT, pre‐surgical vertical STT, emergence angle > 30°, BBT, mPI scores, MT and STH). Multi‐variable analysis confirmed the association of female gender (OR = 4.5, 95% CI: 2.2–9.5, *p* = 0.006), pre‐surgical buccal STT < 1.5 mm (OR = 5.20, 95% CI: 2.09–15.01, *p* = 0.007), mPI scores of 2 (OR = 30.11, 95% CI: 11.00–124.0, *p* < 0.001), BBT (OR = 0.48, 95% CI: 0.31–0.81, *p* = 0.039), MT (OR = 0.20, 95% CI: 0.04–0.39, *p* = 0.001) and STH (OR = 0.50, 95% CI: 0.25–0.89, *p* = 0.002) with peri‐implant mucositis. Multicollinearity led to the exclusion of the mean emergence angle (VIF = 11).

**TABLE 2:** (Top) Logistic regression results on the prevalence of peri‐implant mucositis. (Bottom) Linear regression results on outcome of MRec.

Independent variable	Univariable model			Multivariable model		
	Disease status (peri‐implant mucositis vs. health)					
	OR	Lower–upper (95% CI)	*p*	OR	Lower–upper (95% CI)	*p*
Age	1.00	0.97–1.03	0.84			
Gender (female)	16.00	6.16–46.16	**< 0.001**	4.5	2.2–9.5	**0.006**
Tooth type (molar)	1.75	0.78–3.97	0.17			
Jaw (maxilla)	0.50	0.20–1.15	0.10			
Implant length						
10.5	2.00	0.58–7.98	0.28			
12	0.00	0.00‐inf	0.99			
Implant diameter (4.6)	1.42	0.61–3.34	0.40			
KMW (crown delivery) (< 2)	1.50	0.69–3.77	0.32			
BBT (1 year)	0.54	0.36–0.80	**0.02**	0.48	0.31–0.81	**0.039**
PBT (1 year)	0.66	0.49–3.11	0.119			
Baseline Buccal STT (< 1.5 mm)	12.5	3.33–100.00	**< 0.001**	5.20	2.09–15.01	**0.007**
Baseline vertical STT (< 2 mm)	5.00	2.17–14.29	**0.01**	2.3	0.80–5.50	0.07
Mean emergence angle (> 30°)	1.8	1.4–2.2	**0.04**	Excluded^a^ (VIF = 11)	NA	NA
mPI score						
1	2.57	0.77–10.1	0.141	2.06	0.80–6.90	0.11
2	42.00	10.64–218	**< 0.001**	30.11	11–124	**< 0.001**
STH	0.31	0.15–0.59	**< 0.001**	0.50	0.25–0.89	**0.002**
MT after CD	0.10	0.02–0.33	**< 0.001**	0.20	0.04–0.39	**0.001**

Independent variable	Univariable model			Multivariable model		
	MRec					
	Estimate	SE	*p*	Estimate	SE	*p*
Age	−0.004	0.003	0.194			
Gender (female)	−0.004	0.07	0.562			
Tooth type (molar)	0.167	0.07	**0.03**	0.111	0.05	0.29
Jaw (maxilla)	−0.052	0.08	0.523			
Implant length						
10.5	−0.19	0.11	0.10			
12	−0.14	0.17	0.40			
Implant diameter (4.6)	0.063	0.08	0.45			
KMW (crown delivery) (< 2)	0.41	0.09	**0.01**	0.39	0.07	**0.021**
BBT (1 year)	−0.69	0.09	0.41			
PBT (1 year)	0.87	0.57–1.98	0.67			
Baseline buccal STT (< 1.5 mm)	0.41	0.06	**0.001**	0.27	0.10	**0.03**
Baseline vertical STT (< 2 mm)	0.02	0.12	0.83			
Mean emergence angle (> 30°)	−0.07	0.08	0.36			
mPI score						
1	0.01	0.09	0.89			
2	−0.01	0.10	0.91			
STH	−0.10	0.09	0.25			
MT after CD	−0.45	0.04	**< 0.01**	−0.37	0.06	**< 0.01**

*Note*: Goodness of fit was assessed using pseudo‐*R* ^2^ (0.39) for disease status model and (0.31) for MRec model. (Top) Results of the logistic regression model on the outcome of peri‐implant disease status (Bottom) Results of the linear regression model on the outcome of MRec.

Abbreviations: BBT, buccal bone thickness; CD, crown delivery; KMW, keratinised mucosa width; mPI, modified plaque index; MT, mucosal thickness; PBT, palatal bone thickness; STH, supracrestal tissue height; STT, soft‐tissue thickness.

^a^ Multicollinearity was evaluated using variance inflation factor (VIF), with all included variables having VIF < 10. Mean emergence angle (> 30°) was excluded due to multicollinearity (VIF = 11). The bold values indicate statistical significance.

### 3.3 Factors Affecting MRec and MBL

Table 2 (bottom) and Table S1 present the results of the linear regression on the outcomes MRec and MBL changes, respectively. In the univariable model, several factors were significantly associated with MRec: tooth type, pre‐surgical buccal STT < 1.5 mm, KMW < 2 mm at CD and MT. In multivariable analysis, significant associations with MRec were observed for pre‐surgical buccal STT < 1.5 mm (estimate = 0.27, SE = 0.10, *p* = 0.03), KMW < 2 mm at CD (estimate = 0.39, SE = 0.07, *p* = 0.021) and MT (estimate = −0.37, SE = 0.06, *p* < 0.01).

When it comes to changes in MBL at 5 years, in the univariate analysis, significant associations were observed for KMW < 2 mm at CD, BBT and STH. In the multivariable analysis, KMW < 2 mm at CD (estimate = −0.193, SE = 0.055, *p* = 0.0005) and STH (estimate = −0.366, SE = 0.05, *p* = 0.01) remained significantly associated with MBL changes.

### 3.4 Ultrasonographic Outcomes

Ultrasonographic assessment revealed significantly higher blood flow velocity and perfusion at the mesial, distal and mid‐buccal sites of diseased implants compared to their healthy counterparts (Figure 1, top left). At healthy sites, the blood flow velocity was recorded as 0.071 ± 0.042 (mesial), 0.079 ± 0.041 (mid‐buccal) and 0.080 ± 0.032 (distal), in contrast to 0.263 ± 0.092, 0.224 ± 0.082 and 0.207 ± 0.089, respectively, for peri‐implant mucositis sites. Additionally, the results of the point biserial correlation analysis showed a strong positive association between average ultrasonographic perfusion and disease status (*r* = 0.93, *p* < 0.001) (Figure 2d).

### 3.5 Patient‐Reported Outcomes (Figure 3a–c and Data S1)

**FIGURE 3:**
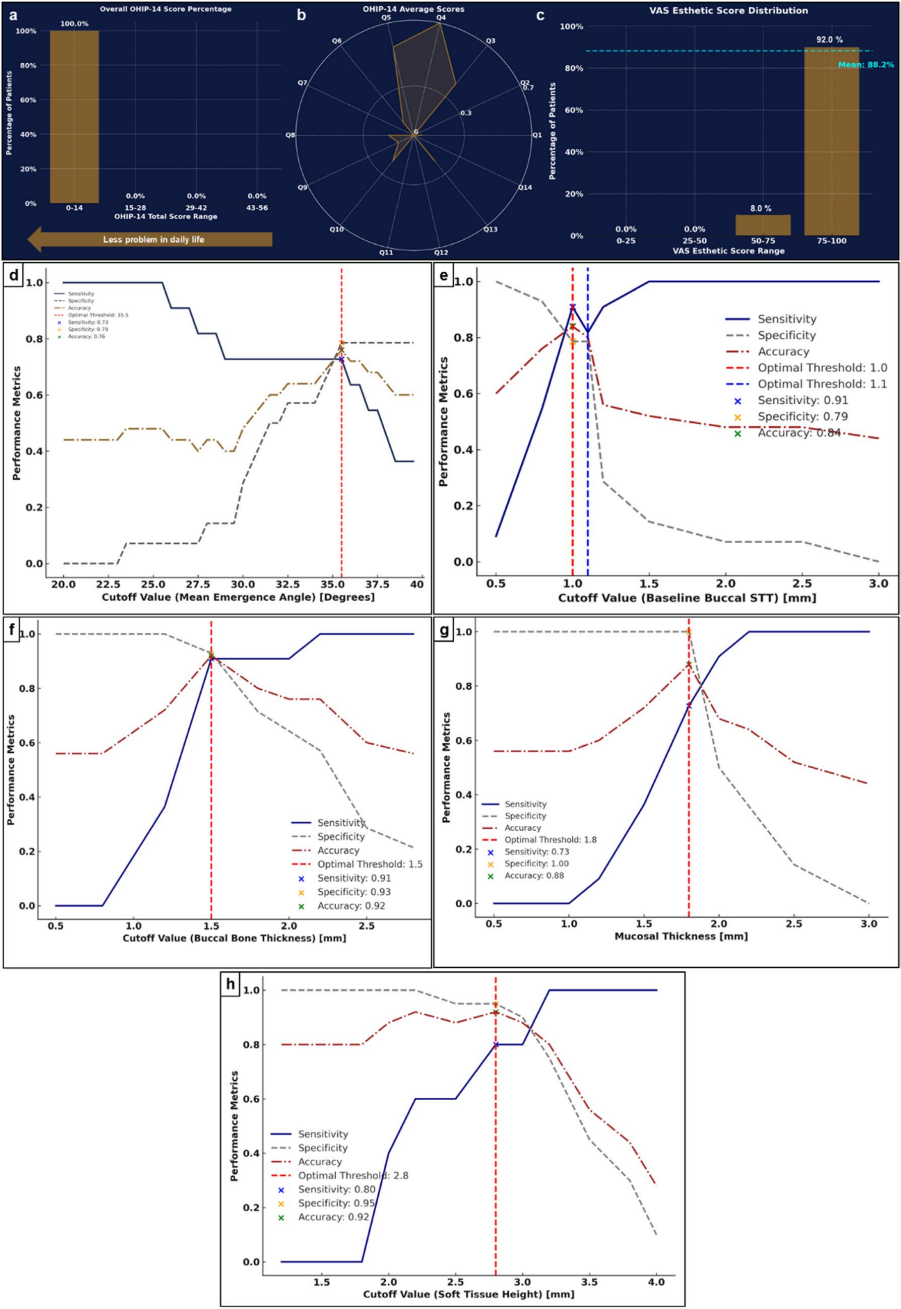
(a–c) PROMs assessed at the 5‐year timepoint of the study. The OHIP‐14 questionnaire results showed that 100% of patients recorded total scores between 0 and 14 points, with 0 indicating no discomfort related to the implant (scale: 0–100). Among the 14 questions in OHIP‐14, questions 4 (discomfort when eating), 5 (self‐conscious because of teeth/implants) and 6 (feeling tense because of tooth/implant problems) demonstrated higher average scores compared to the other domains. Additionally, patient‐reported overall aesthetics and satisfaction, assessed via VAS, yielded an average satisfaction score of 88.2%. The majority of patients (90%, *n* = 23) reported satisfaction levels within the 75%–100% range, while the remaining 8% (*n* = 2) scored between 50% and 75%. Figure 3a–c summarises the PROMs of the study. (a) total OHIP‐14 score and percentage of patients within each score range. (b) Radar plot showing the average scores from each of the OHIP‐14 domains. (c) total aesthetic and satisfaction score from VAS. (d–h) Threshold analysis (via ROC) for significant factors affecting implant health.

### 3.6 Clinical Cut‐Off Values Associated With Long‐Term Implant Health

**FIGURE 4:**
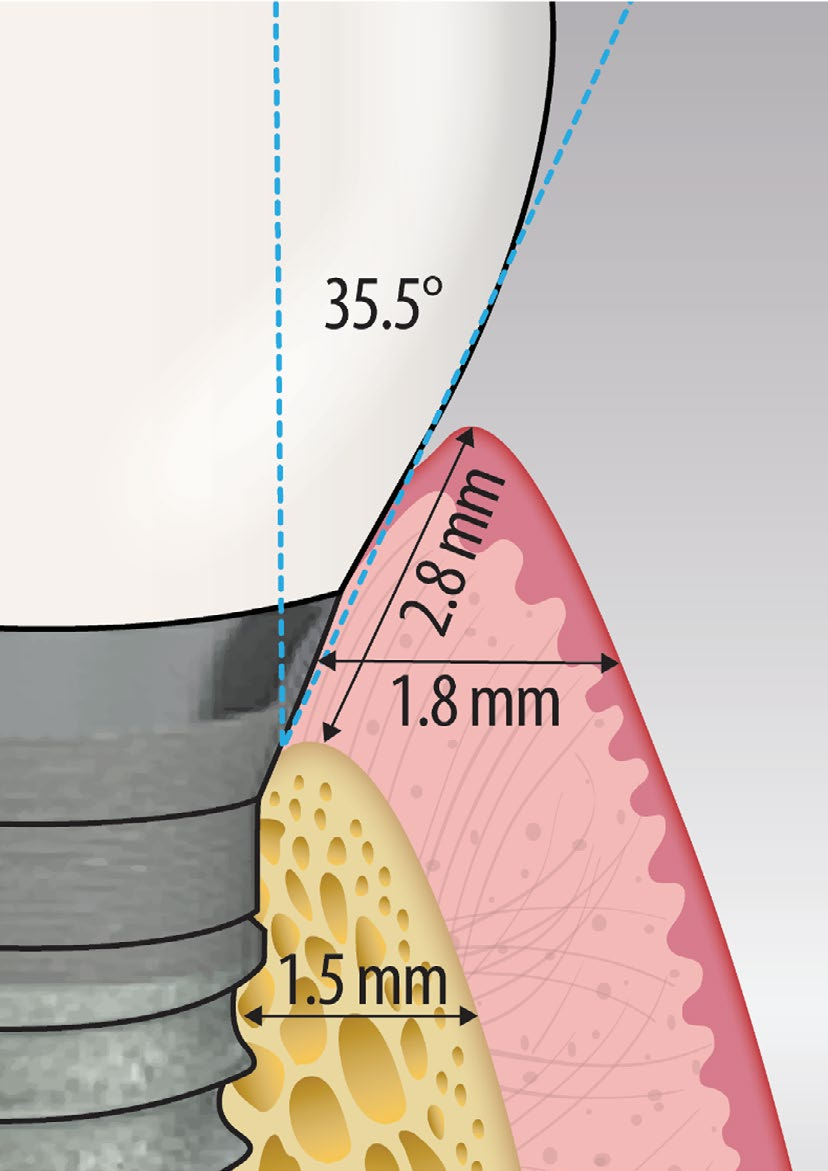
Factors to consider at baseline during STL implant placement and during post‐placement monitoring (restorative emergence angle, STH, MT and BBT).

Receiver operating characteristic (ROC) curve analysis suggested several possible critical clinical cut‐off values associated with maintaining long‐term peri‐implant health (Figure 3d–h). For the restorative emergence angle, a threshold of ≤ 35.5° (sensitivity: 73%, specificity: 79%, accuracy: 76%) was identified. BBT exhibited an optimal threshold of ≥ 1.5 mm (sensitivity: 91%, specificity: 93%, accuracy: 92%). For pre‐surgical buccal STT, a threshold of ≥ 1 mm was determined, showing sensitivity of 91%, specificity of 79% and accuracy of 84%. Similarly, MT at ≥ 1.8 mm demonstrated robust accuracy metrics (sensitivity: 73%, specificity: 100%, accuracy: 88%). Lastly, the optimal threshold for STH was found to be ≥ 2.8 mm (sensitivity: 80%, specificity: 95%, accuracy: 92%) (Figure 4).

## 4 Discussion

### 4.1 Summary of Key Findings

This study assessed the 5‐year outcomes of STL implants placed as part of a prospective cohort study of two groups of ‘thin’ and ‘thick’ peri‐implant soft‐tissue phenotype (Garaicoa‐Pazmino et al. 2021), and explored the relevance of implant‐, site‐ and patient‐related factors with health stability as well as other clinical outcomes. Overall, we noted a 5‐year survival rate of 100%, with 64% of the implants being diagnosed as healthy and 36% presenting with peri‐implant mucositis. While interpretation should take into account the relatively small sample size, the findings provide useful longitudinal data on peri‐implant phenotypical factors over a 5‐year period.

Considering the PROMs and clinical results, which showed an overall satisfaction rate of 88.2%—with no patient reporting more than a minimal functional discomfort—our study supports the medium‐ to long‐term success of the assessed STL implants. A recent retrospective study on 301 STL implants with an average follow‐up of 22.7 ± 14 months by Chacun et al. indicated a survival rate of 98.9% (Chacun et al. 2024). Another similar retrospective study by Schiegnitz et al., reported 96.3% survival rates after 62 ± 31 months follow‐up on tapered STL implants (Schiegnitz et al. 2016). Likewise, Sodnom‐Ish et al., in an up‐to‐5‐year retrospective study assessing tapered, sand‐blasted and acid‐etched surfaced STLs, reported a survival rate of 100% for both 4.0 and 4.5 mm diameter implants and 84.6% for the 5.0 mm diameter STLs (Sodnom‐Ish et al. 2020). While acknowledging differences in implant micro and macro designs, these results align with our study, suggesting a > 95% survival rate for STL implants 5 years post loading (Kim et al. 2018; Roccuzzo et al. 2022).

The primary outcome was peri‐implant disease prevalence. This was assessed clinically using the 2017 AAP/EFP case definitions (Berglundh et al. 2018) and ultrasonographic tissue perfusion for inflammation (Barootchi et al. 2022). In our cohort, several hard‐ and soft‐tissue factors, along with plaque score, were associated with peri‐implant disease (mainly mucositis). These included 1‐year BBT (post loading), baseline STT, STH, MT, higher mPI scores and female gender. Jervøe‐Storm et al. (Jervøe‐Storm et al. 2023) analysed the prevalence of peri‐implant disease in 213 STL implants and reported a rate of 58.7% for peri‐implant mucositis in their 5–17‐year follow‐up cohort. Among other design‐based differences between our study and that of the stated colleagues, this large difference may also be due to the fact that they had solely included patients with a history of stage III/IV periodontitis (Jervøe‐Storm et al. 2023). It should be emphasised that all patients in this cohort were either regular or erratic compliers, with at least one documented supportive peri‐implant therapy visit per year throughout the follow‐up period. While increased plaque levels were significantly associated with mucositis, the consistent participation in maintenance care may have contributed to the absence of peri‐implantitis cases. These findings highlight the potential protective role of supportive care in preserving peri‐implant health. Therefore, the favourable outcomes observed in this study may not be generalisable to populations with poor compliance or complete absence of maintenance therapy.

The 1‐year results of this clinical research indicated no significant difference in terms of MBL changes between thin and thick peri‐implant phenotypes (Garaicoa‐Pazmino et al. 2021). However, when extended to 5 years, our regression analysis revealed a significant association of certain phenotypical components with MBL changes, including KMW at CD (estimate: –0.193, SE: 0.055, *p* < 0.001) and STH (estimate: –0.366, SE: 0.05, *p* = 0.01). These variables were not assessed in the initial 1‐year analysis, precluding a direct comparison of their long‐term impact. In addition, it should be noted that in the current longitudinal assessment, the exact measurements of the soft‐tissue features were used, instead of a binary grouped analysis of thick versus thin.

In a similar study by Spinelli et al. on STL implants similar to those used in the present research, 41 implants placed with a flapless approach showed 100% survival rate and comparable MBL changes (1.10 ± 0.89 mm) (Spinelli et al. 2023). Their findings reported no significant association between MT and MBL changes, contrasting with the results of this study. Instead, a significant association between implant diameter (4.6 mm vs. 3.8 mm) and MBL changes (estimate: −0.7855, SE: 0.3552, *p* = 0.027) was found. These differences may stem from variations in implant placement timing, protocols and patient‐specific factors, such as bone density and mucosal phenotype variability. Notably, the Spinelli study also included three different placement timings, which may account for discrepancies in the findings (Spinelli et al. 2023). The MBL changes from a large sample–sized retrospective assessment by Kang et al. (5‐year: 0.09 ± 0.26 mm and 7‐year: 0.14 ± 0.41 mm) also indicated relatively negligible bone‐level changes in the moderate to long term (Kang et al. 2018). The transgingival healing design of STL implants, used in both studies, may have likely contributed to the observed stability in MBL, which can potentially minimise the risk of microbial contamination and inflammatory responses at the implant–abutment junction by avoiding a subcrestal microgap (Guarnieri et al. 2022). Additionally, studies have shown that STL implants exhibit reduced levels of pro‐inflammatory cytokines, such as interleukin 1‐beta (IL‐1β) and tumoral necrosis factor‐alpha (TNF‐α), which may further support their role in maintaining peri‐implant health (Kim et al. 2010; Menini et al. 2022; Judgar et al. 2014). These findings align with the broader literature, which consistently associates STL implants with favourable outcomes, particularly in terms of MBL stability and reduced peri‐implant complications.

The ultrasonographic peri‐implant tissue perfusion analysis in our study demonstrated a strong positive correlation (*r* = 0.93, *p* < 0.001) with the health status of the implants, suggesting its utility as a marker for peri‐implant inflammation (Barootchi et al. 2022). The observed higher blood flow velocity and perfusion in diseased sites reflect the hypervascularisation commonly associated with inflammation, as blood flow increases to facilitate immune response and tissue repair (Liñares et al. 2015). This observation aligns with histological findings that inflamed peri‐implant tissues exhibit elevated vascularisation and inflammatory markers, as demonstrated by increased expression of IL‐1β and TNF‐α in studies on peri‐implant soft tissue (Liñares et al. 2015). Additionally, the heightened perfusion could be linked to the inflammatory cell infiltrates and vascular changes often reported in the vicinity of implants with microbial biofilms or compromised soft‐tissue integration (Guarnieri et al. 2022). These findings align with the role of vascular activity in the progression of peri‐implant diseases, highlighting ultrasonography as a valuable, non‐invasive modality for assessing peri‐implant tissue health (Barootchi et al. 2022; Galarraga‐Vinueza et al. 2024). Previous studies have supported the use of ultrasound to evaluate soft‐tissue integration and vascular characteristics (Barootchi et al. 2022; Galarraga‐Vinueza et al. 2024), emphasising its potential to complement conventional clinical and radiographic assessments.

### 4.2 Strengths and Limitations

The strengths of this study include its longitudinal 5‐year follow‐up, very low dropout rate and comprehensive evaluation of the peri‐implant phenotype through clinical, radiographic and ultrasonographic assessments. Examiner blinding and calibration enhanced methodological rigour, and incorporating PROMs provided a broader perspective on treatment success beyond conventional clinical measures.

However, a few limitations of this study must be acknowledged. Despite our low dropout rate in the medium‐ to long‐term follow‐up, the relatively small sample size of the original research might have limited the statistical power and generalisability of our findings. Thus, we encourage external validation and future research in other cohorts to verify our results. Additionally, the use of a single implant type restricts the applicability of our results to other implant systems. Although several measures were taken to minimise model complexity and reduce collinearity, some risk of overfitting remains, which is due to the limited sample size. It should also be noted that the emergence angle was excluded from the final multivariable model due to high collinearity, despite showing a strong univariable association with disease prevalence and a clearly defined threshold in ROC analysis. Its retention in the discussion reflects its potential clinical relevance; however, the absence from the adjusted model means that this factor should be interpreted with caution until validated in larger and more diverse datasets. Furthermore, the 2D approach for evaluating the emergence profile carries inherent limitations. The analysis assumes a consistent emergence angle circumferentially around the crown, while this angle may vary substantially between the buccal and lingual aspects. This simplification could underestimate 3D variations that may be clinically relevant. These highlight the need for future studies with larger samples, varied implant systems and additional biomarkers to validate these findings.

### 4.3 Interpretation and Implications

Taken together, the results highlight the importance of peri‐implant phenotype and implant design in treatment planning. STL implants address key biological and mechanical challenges by reducing microgap‐associated bacterial colonisation and crestal bone loss; Hermann et al. reported ~2 mm of bone loss around two‐piece implants due to microgap dynamics (Hermann et al. 2001). With transgingival healing, STL implants avoid abutment manipulation, preserve biological width and improve soft‐tissue stability. They also enable single‐stage protocols, reducing patient discomfort, treatment time and mucosal trauma (Barrachina‐Díez et al. 2013). Overall, this design promotes soft‐tissue adaptation, minimises inflammation and ensures stable outcomes with improved patient satisfaction.

Based on the longitudinal assessment of STL implants in our study, the following potential clinical considerations can be presented:

1. The assessment of factors associated with peri‐implant health suggests several values to consider when placing STL implants. A mean restorative emergence angle of ≤ 35.5°, BBT of ≥ 1.5 mm, pre‐surgical buccal STT of ≥ 1 mm, STH of ≥ 2.8 mm and MT of ≥ 1.8 mm were associated with a healthy implant status in this cohort. While causality cannot be inferred, these values may serve as reference points for clinical monitoring or future research.
2. Several factors are encouraged to be considered especially when placing STL implants in the aesthetic zone, as they were associated with a higher risk of developing MRec and peri‐implant soft‐tissue deficiencies: KMW (2 mm), baseline buccal STT and post‐operative MT.
3. Clinicians may consider the identified associations when planning implant therapy, particularly in regard to soft‐tissue phenotype and prosthetic contours, while recognising the exploratory nature of these findings.

## 5 Conclusions

This 5‐year longitudinal cohort analysis on soft tissue–level implants found 100% survival rate and high patient satisfaction (88.2%). Several peri‐implant parameters, including pre‐surgical buccal STT (≥ 1 mm), KMW (≥ 2 mm), STH (≥ 2.8 mm) and BBT (≥ 1.5 mm), showed significant associations with peri‐implant health and stability over time. MBL changes were minimal and clinically acceptable, which may support the favourable outcomes of tissue‐level implants' transgingival healing design. While these findings suggest potential clinical relevance, they should be interpreted cautiously within the limitations of the study design, and further research in larger and more diverse populations is needed to validate these associations.

## Author Contributions

H.S.: conceptualisation, data collection, ultrasonographic analysis, statistical analysis and manuscript writing. P.H.: data collection, radiographic analysis and manuscript writing. L.T., C.G.P.: conceptualisation and data collection. J.C.: conceptualisation, data collection and critical review. H.‐L.W.: conceptualisation, surgical intervention, data collection, surgical phase, radiographic analysis, manuscript writing and critical review. S.B.: conceptualisation, ultrasonographic analysis, manuscript writing and critical review. All authors gave final approval for the version to be published.

## Funding

The authors received no specific funding for this work.

## Conflicts of Interest

Dr. Hom‐Lay Wang occasionally speaks for BioHorizons Inc. (whose implants were used in this study) and receives honoraria for this service. The other authors declare no conflicts of interest.

## Supporting information


**Data S1:** Supporting Information.

## Data Availability

The data that support the findings of this study are available from the corresponding author upon reasonable request.
