# Supplementary material for: Impact of Peri‐Implant Phenotype on Implant Therapy Outcomes: A 5‐Year Cohort Analysis on Soft Tissue—Level Implants
Source: J Clin Periodontol. 2025 Dec 12;53(3):394–406. doi: 10.1111/jcpe.70062 (PMC12890457; doi:10.1111/jcpe.70062)
Supplement: Supplementary file 1 — Data S1: Supporting Information. [file JCPE-53-394-s001.docx]

**Supplementary Materials**

**Impact of peri-implant phenotype on implant therapy outcomes: A 5-year cohort analysis on soft tissue-level implants**

Hamoun Sabri, Parham Hazrati, Lorenzo Tavelli, Carlos Garaicoa-Pazmino, Javier Calatrava, Hom-Lay Wang, and Shayan Barootchi

Contents

[**Interventions** 2](#_Toc198397254)

[Supportive care protocols 3](#_Toc198397255)

[Eligibility criteria 3](#_Toc198397256)

[Clinical variables 4](#_Toc198397257)

[Ultrasonographic Assessment 4](#_Toc198397258)

[**Calibration protocol** 5](#_Toc198397259)

[**Patient reported outcomes** 6](#_Toc198397260)

[**Statistical analysis** 7](#_Toc198397261)

[Results 8](#_Toc198397262)

[**Descriptive statistics and study population** 8](#_Toc198397263)

[Limitations 10](#_Toc198397264)

[Supplementary figures and tables 10](#_Toc198397265)

[**Appendix Table 1.** 10](#_Toc198397266)

[**Appendix Figure 1.** 11](#_Toc198397267)

[STROBE Statement 12](#_Toc198397268)

**Methods**

## **Interventions**

Implant surgical procedures were performed by a single operator (HL-W) under local anesthesia with 2% lidocaine containing 1:100,000 epinephrine, with an additional 2% lidocaine containing 1:50,000 epinephrine for hemostasis. Intrasulcular incisions were made around the teeth adjacent to the edentulous site, along with a mid-crestal incision bisecting the keratinized mucosa. Full-thickness buccal and lingual/palatal flaps were elevated to expose the alveolar ridge. Measurements of pre-surgical buccal STT (at 3 mm apical to the incision line) and pre-surgical vertical STT (center of the ridge) were recorded intraoperatively using a dental caliper and a periodontal probe.

Osteotomies were prepared according to the manufacturer’s drilling sequence using a surgical guide, and implants were placed with the smooth-rough junction positioned at the alveolar crest (Tapered Tissue Level implant, BioHorizons, Birmingham, AL.). Implant diameter (3.8 mm or 4.5 mm) and length (9 mm, 10.5 mm and 12 mm) were selected based on ridge width and anatomical considerations. Healing abutments (4-mm height, regular emergence profile) were seated immediately after placement, and surgical sites were closed with interrupted dense polytetrafluoroethylene sutures. A standardized periapical radiograph was also obtained postoperatively.

Postoperative care included a 2-week course of warm saltwater rinses, amoxicillin 500 mg three times daily for 10 days (or azithromycin for those with allergy to beta-lactams), and ibuprofen 600 mg for pain management. Sutures were removed 2 weeks postoperatively, with follow-up visits at 1 and 4 months to ensure proper healing.

Prosthetic treatment was initiated 3 to 5 months after implant placement, with custom abutments and screw-retained prostheses delivered 2 to 4 weeks post-impression. Occlusion, crown contours, and margin seals were evaluated and adjusted as needed.

# **Supportive care protocols**

Based on available recall records and clinical documentation, supportive peri-implant therapy (SPIT) compliance was estimated and categorized as follows: non-compliance (no SPIT visits during the 5-year follow-up), erratic compliance (approximately 1 SPIT/year), and regular compliance (≥2 SPIT)^1^.

# **Eligibility criteria**

The original patient selection criteria required participants to meet all of the following inclusion criteria: (1) aged >18 years; (2) partially edentulous in the maxillary or mandibular premolar or first molar region; (3) presence of adjacent teeth mesial and distal to the edentulous site; (4) residual bone height >9 mm and bone width >5 mm; (5) >2 mm KMW (at the time of screening); (6) optimal oral hygiene, defined as full-mouth plaque scores <10%; and (7) clinical gingival health on an intact or reduced periodontium. Exclusion criteria included: (1) need for bone augmentation; (2) current smoking or smoking cessation within <1 year; (3) current or planned pregnancy; (4) uncontrolled systemic disease; (5) conditions affecting bone metabolism (e.g., diabetes, osteopenia, osteoporosis, hyperparathyroidism); (6) current or historical use of oral or intravenous bisphosphonates; (7) history of radiation therapy; (8) need for active periodontal therapy.

# **Clinical variables**

- **KMW:** This was assessed on the mid-buccal aspect of the implant, from mucosal margin to the mucogingival junction.
- **Modified plaque index (mPI):** Based on a 0-3 scale^2^, measurements were collected on the buccal, mesial, distal and lingual sites of each implant, and the highest score per implant was recorded.

**Bleeding upon probing (BoP) and suppuration (SUP):** BoP was determined using a scale of 0-2 (0: no BoP, 1: bleeding spot, 2: profuse bleeding). Additionally, SUP was assessed as a binary outcome (presence/absence) upon probing.

# **Radiographical assessment**

Standardized intra-oral peri-apical radiographs were obtained using an x-ray stent that had been fabricated at the baseline assessment of the subjects. Additionally, a cone beam computed tomography (CBCT) scan with the same setup and features of the initial study phase^21^ was obtained. MBL measurements were obtained from 2D standardized radiographs on both the mesial and distal sides of each implant using a commercially available software (ImageJ, National Institutes of Health, Bethesda, USA). The mean MBL value for each implant was calculated based on the distance from the implant platform to the bone-to-implant contact (BIC). For each implant, the average of mesial and distal measurements was taken as the mean MBL, and the difference between two time points was calculated as the MBL changes. Radiographic examinations were performed by one calibrated examiner on standardized intraoral periapical images. Measurement error was assessed as the absolute difference^27^ between repeated assessments on 10 non-study implant radiographs, resulting in a mean error of 0.19±0.21 mm and intra-examiner ICC of 0.92 (95% CI: 0.88—0.96).

Additionally, the restoration emergence angle was measured on the mesial and distal of each implant as described before^24^, and a mean value was defined based on these for each subject.

On a 3D assessment (CBCT), the buccal and palatal bone thickness (BBT, PBT) at 2-, 4- and 6-mm below the implant platform was assessed in mms using DICOM processing software (Blue Sky, Blue Sky Bio LLC, Grayslake, IL). A mean value of three measurements for each variable was considered as the total BBT and PBT. **Figure 1 (bottom)** depicts the radiographical assessment performed.

# **Ultrasonographic Assessment**

A pre-calibrated blinded examiner performed ultrasonographic image acquisition (S.B) (the calibration phase protocol is provided **below**). The complete ultrasonographic assessment protocol has been described in previous publications.^3,4^ on the mid-facial ultrasound scans the following variables were measured **(figure 1; top right.** Briefly, a mid-facial scan was obtained from each implant site on B-Mode (2D cross-sectional anatomical grayscale images) and the Color-doppler mode positioning the ultrasound probe/tranducer in a way that its long axis was parallel to the long axis of the implant fixture **(figure 1; top left)**. The displayed color velocity (CV) presents the projection of the actual velocity onto the ultrasound beam. This is determined by the measured mean phase shift, which is mathematically equivalent to the true mean velocity within the point-spread function multiplied by the cosine of the angle to the ultrasound beam. This process visualizes the velocity of blood flow within the lumens in the field of view. In addition to the blood flow assessment, the following peri-implant parameters were also collected from the mid-facial ultrasound scans **(Figure 1; top right)**:

- **MT:** measured at 1-,3-, and 5-millimeters below the mucosal margin. A mean value was considered for each implant for the analysis. This corresponds to soft tissue thickness around implants after implant restoration.
- **STH:** as the linear distance from mucosal margin to the bone crest^5^. This corresponds to the vertical supracrestal tissue around implants after prosthesis delivery.

## **Calibration protocol**

It should be noted that examiners who were involved in the collection of clinical, radiographic, and ultrasonographic data were blinded to the initial treatment allocation of each patient.

The ultrasonographic images were obtained by one calibrated examiner with at least 5 years of experience in periodontal and implant ultrasonography (S.B). Prior to initiation of study visits, the examiner underwent a calibration process over a 2-week period using 5 non-study subjects and a total of 10 sites. Mucosal thickness was measured at 1 mm, 3 mm, and 5 mm apical to the mucosal margin. Two sets of repeated measurements were performed one week apart. An intra-examiner reliability of at least 90% was aimed for and successfully achieved.

Radiographic measurements were performed by a newly assigned examiner (P.H) who analyzed standardized periapical radiographs and CBCT scans. Calibration was conducted over a 2-week period on 10 non-study implants, in which buccal and palatal bone thickness as well as MBLs were repeatedly measured. Intra-examiner reliability testing demonstrated a concordance value of 92%.

Clinical parameters at baseline and 1-year follow-ups—including PPD, BoP, MRec, KMW and plaque score—were recorded by a single calibrated examiner in the original trial. However, due to the unavailability of the same examiner at the 5-year follow-up, a new examiner performed the clinical recordings (S.B). Calibration for this examiner was conducted over a 2-week period on 5 non-study subjects, following the same protocol, and intra-examiner reliability of 94% was achieved.

## **Patient reported outcomes**

At the end of the 5-year follow-up visits, the PROMs were assessed using a standardized questionnaire filled out by patients. The PROMs assessed consisted of:

- A Visual Analogue Scale (VAS) [0-100] corresponding to overall aesthetic and satisfaction of the soft tissue around implant.
- Patient-reported concerns regarding each implant including aesthetics, sensitivity to temperature fluids, sensitivity to brushing, development of an issue or problem and fear of losing the implant. Each of these domains were scored from 0 (no concern) to 4 (lots of concern).
- OHIP-14 questionnaire was used to assess the inconvenience/problems patient experiences in daily life with regard to oral health and function. This consisted of 14 questions with a score of 0 (never) to 4 (very often), with the higher score (closer to the total of 56) indicating more functional problems and disabilities. Mainly, the questions address function, phonetics, mastication, psychological, diet, social life and overall quality of life satisfaction (related to oral health).

## **Statistical analysis**

Descriptive statistics were used to summarize continuous variables as means, medians, standard deviations, and ranges, while categorical variables were expressed as frequencies and percentages. Normality of data distribution was assessed using the Shapiro-Wilk test. Based on the results, paired t-tests were used for parametric comparisons, while the Wilcoxon signed-rank test was employed for non-parametric data. A logistic regression model was used to analyze the primary outcome of peri-implant disease status, while linear regression models were applied for secondary outcomes, including changes in MBL and MRec. Standard logistic and linear regression models were used, as each patient contributed a single implant and no clustering was present. Variables with significance in the univariate analysis were entered into stepwise regression, followed by multi-variable regression. Multicollinearity was assessed in multi-variable models using the Variance Inflation Factor (VIF), and variables with VIF > 10 were excluded. Model fit was evaluated using pseudo-R² values.

As a post-hoc analysis, variables significantly associated with peri-implant health outcomes were subjected to Receiver Operating Characteristic (ROC) curve analysis to determine optimal clinical thresholds. These thresholds were identified using the Youden index, and sensitivity, specificity, accuracy, and Youden index values were calculated to evaluate the clinical performance of each threshold.

PROMs were analyzed descriptively, with results presented using medians, interquartile ranges, and proportions as appropriate.

To account for multiple testing, all p-values were adjusted using the False Discovery Rate (FDR) method. A two-tailed p-value < 0.05 was considered statistically significant for all analyses. The statistical analysis was performed by one investigator (H.S) using RStudio and the following packages: lme4, glm, stats, pROC, ggplot2, and car. As this study represents a 5-year longitudinal follow-up of the original controlled trial, no new sample size calculation was performed; the analysis was conducted per protocol based on the original cohort, with only one dropout during follow-up. For transparency, a sub-analysis was also performed to descriptively and statistically compare thin versus thick baseline mucosal thickness groups, which is presented in **Supplementary Table 2.**

# **Results**

## **Descriptive statistics and study population**

The patient cohort included 15 males (60%) and 10 females (40%) with a mean age of 54.20±12.01 years. Implants were placed in 9 maxillary (36%) and 16 mandibular sites (64%), with 11 implants (44%) placed in premolar and 14 implants (56%) in molar regions. The majority of implants had a length of 10.5 mm (80%), while 9 mm and 12 mm implants accounted for 12% and 8% of placements, respectively. Regarding implant diameter, 17 implants (68%) were 3.8 mm, and 8 implants (32%) were 4.6 mm. Among the 25 included patients, 14 were classified as erratic compliers and 11 as regular compliers based on SPIT attendance patterns over the 5-year period. All patients received at least one maintenance visit per year, and none were non-compliant. **Table 1** presents the characteristics of the included subjects and implants. The mesial and distal emergence angle of the implant restorations were 36.88±7.21 and 35.19±10.98 degrees respectively with an average of 36.04±8.41 degrees.

When it comes to the baseline characteristics, a mean pre-surgical buccal STT of 0.84±0.39mm and vertical STT of 2.16±0.53 mm was recorded. The MT around restored implants was 1.87±0.40mm while STH was 3.26±0.70mm.

Over the 5-year follow-up period, clinical variables were recorded at different time points (**Table 1 and Figure 2a-c**). MBLs were 0.78±0.38 mm at baseline (BL), 1.01±0.47 mm at crown delivery (CD), 1.28±0.42 mm at 1 year (1Y), and 1.57±0.42 mm at 5 years (5Y), with changes of 0.23±0.22 mm [0–0.9] between BL and CD, 0.27±0.25 mm [0.09–1.09] between CD and 1Y, and 0.56±0.39 mm [0.06–1.33] from CD to 5Y. PPD increased from 2.31±0.41 mm at CD to 3.26±0.49 mm at 1Y and 3.96±0.51 mm at 5Y, with statistically significant changes of 0.95±0.65 mm [0.33–2.17] between CD and 1Y and 1.65±0.63 mm [0.63–3.03] from CD to 5Y. BBT was 1.88±0.57 mm at 1Y and 1.84±0.72 mm at 5Y, with a change of -0.03±0.81 mm between 1Y and 5Y. PBT was 2.17±0.92 mm at 1Y and 2.36±0.95 mm at 5Y, with a change of 0.19±0.07 mm between 1Y and 5Y. KTW was 2.76±1.06 mm at CD, 2.88±1.00 mm at 1Y, and 3.04±1.54 mm at 5Y, with a change of 0.27±1.34 mm from CD to 5Y. MRec was 0.00±0.00 mm at CD and 1Y and 0.26±0.54 mm at 5Y, with no significant changes over time. Statistically significant differences were observed only for PPD changes between CD and 1Y and CD and 5Y.

**3.2. Peri-implant disease and associated factors (Univariable models results)**

The univariable model in outcome of peri-implant disease status indicated several significant factors. These included gender (female: OR=16.00, 95% CI: 6.16–46.16, p<0.001), baseline buccal STT <1.5mm (OR=12.5, 95% CI: 3.33–100.0, p<0.001), baseline vertical STT <2mm (OR=5.00, 95% CI: 2.17–14.29, p=0.01), MRec over 5 years (OR=7.50, 95% CI: 2.10–16.80, p=0.01) and emergence angle >30 degrees (OR=1.8, 95% CI: 1.4-2.2, p=0.04) as well as BBT at 1-year (OR= 0.54, 95% CI: 0.36–0.80, p=0.02). Additionally, mPI scores of 2 (OR=42.0, 95% CI: 10.64–218, p<0.001), mucosal thickness (OR=0.10, 95% CI: 0.02–0.33, p<0.001), and STH (OR=0.31, 95% CI: 0.15–0.59, p<0.001) also showed significant associations. The result of multivariable model is provided in the main manuscript.

In the univariable analysis on outcome of MRec, it significantly associated with the tooth type (molar: estimate=0.167, SE=0.07, p=0.03), pre-surgical buccal STT <1.5mm (estimate=0.41, SE=0.06, p=0.001), KMW at CD <2mm (estimate=0.41, SE=0.09, p=0.01), and MT (estimate=-0.45, SE=0.04, p<0.01).

When it comes to changes in MBL at 5 years, in the univariate analysis, significant associations with MBL changes were observed for KMW at CD <2mm (estimate=-0.214, SE=0.055, p<0.001), BBT at 1 year (estimate=-0.18, SE=0.071, p=0.03 and STH (estimate=-0.466, SE=0.152, p=0.005). The results of multivariable models on outcomes of MRec and MBL changes are also described in the main manuscript.

**Subgroup analysis**

For transparency regarding the initial study design, descriptive and inferential statistics were stratified by baseline vertical STT (thin ≤2 mm vs. thick >2 mm). As shown in **Appendix Table 2**, changes in MBL, buccal and palatal bone thickness, PPD, KMW, MRec, and PROMs were compared between groups. While mean values tended to show slightly greater bone and soft tissue changes in the thin group (e.g., higher MBL change and mucosal recession), none of these differences reached statistical significance after FDR adjustment (all p > 0.05).

# Supplementary figures and tables

**Appendix Table 1.** Results of the linear regression model on the outcome of MBL changes. (KMW: keratinized mucosa width, BBT: buccal bone thickness, PBT: palatal bone thickness, STT: soft tissue thickness, STH: Supracrestal tissue height, MT: Mucosal thickness, mPI: modified plaque index, CD: Crown delivery)

|  | **Univariate Models** | | | **Multivariable Model** | | |
| --- | --- | --- | --- | --- | --- | --- |
|  | **MBL changes** | | | | | |
| **Independent variable** | **Estimate** | **SE** | **P Value** | **Estimate** | **SE** | **P Value** |
| **Age** | -0.004 | 0.006 | 0.518 |  |  |  |
| **Gender (Female)** | 0.009 | 0.157 | 0.956 |  |  |  |
| **Tooth Type (Molar)** | 0.080 | 0.154 | 0.607 |  |  |  |
| **Jaw (Maxilla)** | -0.107 | 0.159 | 0.506 |  |  |  |
| **Implant Length** |  |  |  |  |  |  |
| 10.5 | -0.380 | 0.229 | 0.111 |  |  |  |
| 12 | -0.235 | 0.337 | 0.493 |  |  |  |
| **Implant Diameter (4.6)** | -0.043 | 0.164 | 0.795 |  |  |  |
| **KMW (Crown delivery) (<2)** | -0.214 | 0.05535 | **<0.001** | -0.193 | 0.055 | **0.0005** |
| **BBT (1 Year)** | -0.18 | 0.071 | **0.03** | -0.11 | 0.09 | 0.27 |
| **PBT (1 Year)** | 0.0002 | 0.0817 | 0.98 |  |  |  |
| **Baseline Buccal STT (<1.5mm)** | 0.161 | 0.234 | 0.498 |  |  |  |
| **Baseline Vertical STT (<2mm)** | -0.291 | 0.228 | 0.215 |  |  |  |
| **Mean Emergence Angle (>30 degrees)** | 0.038 | 0.171 | 0.827 |  |  |  |
| **mPI Score** |  |  |  |  |  |  |
| 1 | -0.253 | 0.186 | 0.187 |  |  |  |
| 2 | -0.109 | 0.195 | 0.582 |  |  |  |
| **STH** | -0.466 | 0.152 | **0.005** | -0.366 | 0.05 | **0.01** |
| **MT after CD** | -0.075 | 0.164 | 0.652 |  |  |  |

*Goodness-of-fit was assessed using Pseudo-R² (0.29).

*Multicollinearity was evaluated using Variance Inflation Factor (VIF), with all included variables having VIF < 10.

**Appendix Table 2.** Descriptive statistics stratified by the initial study group (based on the 1-year study cohort groups; Thin (≥2mm) and thick (<2mm) baseline vertical soft tissue). [CD: crown delivery, MBL: Marginal bone level, KMW: keratinized mucosa width, MRec: mucosal recession, VAS: visual analogue scale, SD: standard deviation]

|  | | **Group** | | |  |
| --- | --- | --- | --- | --- | --- |
| **Variable** | | **Thin (mean [SD])** | **Thick (mean [SD])** | **Overall (mean [SD])** | **P Value*** |
| **MBL Change – 1 to 5 Years** | | 0.41 (0.36) | 0.17 (0.16) | 0.29 (0.29) | 0.134 |
| **MBL Change - CD to 5 Years** | | 0.69 (0.44) | 0.43 (0.31) | 0.56 (0.39) | 0.201 |
| **Buccal Bone Thickness Change** | -0.05 (0.74) | | -0.03 (1.10) | -0.03 (0.81) | 0.946 |
| **Palatal/lingual Bone Thickness Change** | | -0.00 (0.45) | 0.41 (0.53) | 0.19 (0.07) | 0.957 |
| **PPD Change – CD to 5 Years** | | 1.26 (0.47) | 1.56 (0.46) | 1.65 (0.63) | 0.221 |
| **KMW Change** | | 0.30 (2.07) | 0.25 (1.62) | 0.27 (1.34) | 0.218 |
| **MRec (CD to 5 Years)** | | 0.39 (0.27) | 0.14 (0.73) | 0.26 (0.54) | 0.218 |
| **VAS Esthetic Score** | | 85.08 (10.94) | 94.69 (5.24) | 90.08 (9.62) | 0.113 |

*****Between group comparison based on Wicoxon Signed-rank test or paired t-test and Benjamini-Hochberg FDR-adjusted P values.

**Appendix Figure 1.** Study flow-chart, including initial phase (1-year) and longitudinal assessment (5-years). Note that one patient dropped out between the 1-year and 5-year follow-ups due to unwillingness to undergo a CBCT scan.

**
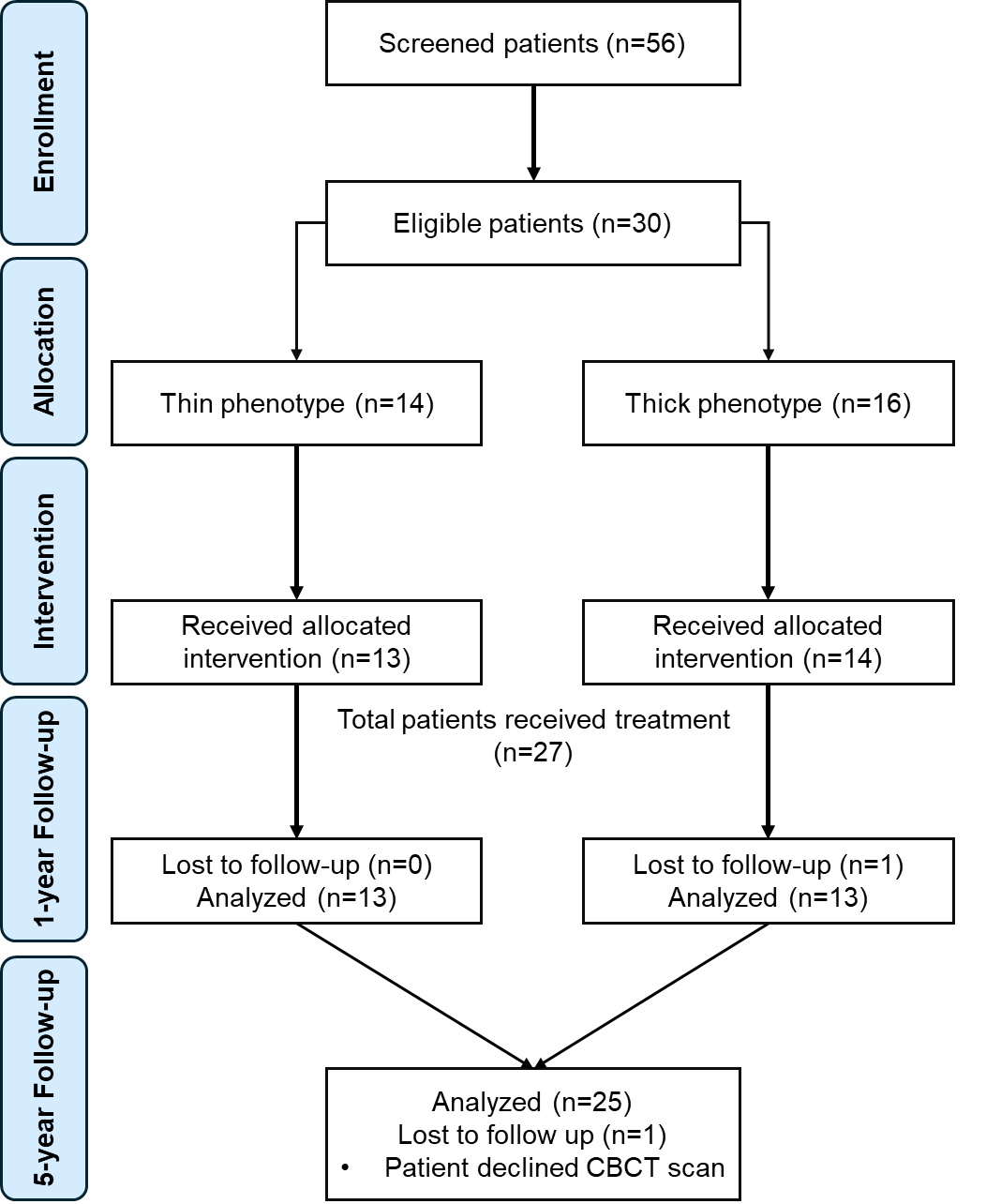
**

**STROBE Statement**—Checklist of items that should be included in reports of ***cross-sectional studies***

|  | Item No | Recommendation | Section/page number |
| --- | --- | --- | --- |
| **Title and abstract** | 1 | (*a*) Indicate the study’s design with a commonly used term in the title or the abstract | 1 |
|  |  | (*b*) Provide in the abstract an informative and balanced summary of what was done and what was found | 1 |
| Introduction | | |  |
| Background/rationale | 2 | Explain the scientific background and rationale for the investigation being reported | 2-3 |
| Objectives | 3 | State specific objectives, including any prespecified hypotheses | 3 |
| Methods | | |  |
| Study design | 4 | Present key elements of study design early in the paper | 3-4 |
| Setting | 5 | Describe the setting, locations, and relevant dates, including periods of recruitment, exposure, follow-up, and data collection | 3 |
| Participants | 6 | (*a*) Give the eligibility criteria, and the sources and methods of selection of participants | 3 |
| Variables | 7 | Clearly define all outcomes, exposures, predictors, potential confounders, and effect modifiers. Give diagnostic criteria, if applicable | 4-5 |
| Data sources/ measurement | 8* | For each variable of interest, give sources of data and details of methods of assessment (measurement). Describe comparability of assessment methods if there is more than one group | *5-6* |
| Bias | 9 | Describe any efforts to address potential sources of bias | Appendix |
| Study size | 10 | Explain how the study size was arrived at | 5-6 |
| Quantitative variables | 11 | Explain how quantitative variables were handled in the analyses. If applicable, describe which groupings were chosen and why | 6-7 |
| Statistical methods | 12 | (*a*) Describe all statistical methods, including those used to control for confounding | Appendix |
|  |  | (*b*) Describe any methods used to examine subgroups and interactions | Appendix |
|  |  | (*c*) Explain how missing data were addressed | Appendix |
|  |  | (*d*) If applicable, describe analytical methods taking account of sampling strategy | Appendix |
|  |  | (*e*) Describe any sensitivity analyses | Appendix |
| Results | | |  |
| Participants | 13* | (a) Report numbers of individuals at each stage of study—eg numbers potentially eligible, examined for eligibility, confirmed eligible, included in the study, completing follow-up, and analysed | Appendix |
|  |  | (b) Give reasons for non-participation at each stage | Supplementary figure |
|  |  | (c) Consider use of a flow diagram | Supplementary figure |
| Descriptive data | 14* | (a) Give characteristics of study participants (eg demographic, clinical, social) and information on exposures and potential confounders | Appendix |
|  |  | (b) Indicate number of participants with missing data for each variable of interest | Appendix |
| Outcome data | 15* | Report numbers of outcome events or summary measures | 5-7 |
| Main results | 16 | (*a*) Give unadjusted estimates and, if applicable, confounder-adjusted estimates and their precision (eg, 95% confidence interval). Make clear which confounders were adjusted for and why they were included | 12 |
|  |  | (*b*) Report category boundaries when continuous variables were categorized | NA |
|  |  | (*c*) If relevant, consider translating estimates of relative risk into absolute risk for a meaningful time period | NA |
| Other analyses | 17 | Report other analyses done—eg analyses of subgroups and interactions, and sensitivity analyses | NA |
| Discussion | | |  |
| Key results | 18 | Summarise key results with reference to study objectives | 13-14 |
| Limitations | 19 | Discuss limitations of the study, taking into account sources of potential bias or imprecision. Discuss both direction and magnitude of any potential bias | Appendix |
| Interpretation | 20 | Give a cautious overall interpretation of results considering objectives, limitations, multiplicity of analyses, results from similar studies, and other relevant evidence | 13-14 |
| Generalisability | 21 | Discuss the generalisability (external validity) of the study results | 14 |
| Other information | | |  |
| Funding | 22 | Give the source of funding and the role of the funders for the present study and, if applicable, for the original study on which the present article is based | Title page |

**References**

1. Monje A, Wang HL, Nart J. Association of Preventive Maintenance Therapy Compliance and Peri-Implant Diseases: A Cross-Sectional Study. *J Periodontol*. Oct 2017;88(10):1030-1041. doi:10.1902/jop.2017.170135

2. Mombelli A, van Oosten MA, Schurch E, Jr., Land NP. The microbiota associated with successful or failing osseointegrated titanium implants. *Oral Microbiol Immunol*. Dec 1987;2(4):145-51. doi:10.1111/j.1399-302x.1987.tb00298.x

3. Barootchi S, Tavelli L, Majzoub J, Chan HL, Wang HL, Kripfgans OD. Ultrasonographic Tissue Perfusion in Peri-implant Health and Disease. *J Dent Res*. Mar 2022;101(3):278-285. doi:10.1177/00220345211035684

4. Galarraga-Vinueza ME, Barootchi S, Mancini L, Sabri H, Schwarz F, Gallucci GO, Tavelli L. Echo-intensity characterization at implant sites and novel diagnostic ultrasonographic markers for peri-implantitis. *J Clin Periodontol*. Apr 1 2024;doi:10.1111/jcpe.13976

5. Avila-Ortiz G, Gonzalez-Martin O, Couso-Queiruga E, Wang HL. The peri-implant phenotype. *J Periodontol*. Mar 2020;91(3):283-288. doi:10.1002/jper.19-0566
